# Comparison of activation and selectivity in dorsal and ventral epidural spinal cord stimulation in rats: a computational modeling study

**DOI:** 10.1038/s41598-025-19555-w

**Published:** 2025-10-13

**Authors:** Dinglong Yan, Zheshan Guo, Haipeng Liu, Xiao Wang, Fengyan Liang, Jing Jie, Ming Yin

**Affiliations:** 1 State Key Laboratory of Digital Medical Engineering, Key Laboratory of Biomedical Engineering of Hainan Province, Sanya Research Institute of Hainan Univiersity, School of Biomedical Engineering, Hainan University, Sanya, 572024 P R China; 2 Centre for Intelligent Healthcare, Coventry University, Coventry, UK; 3 National Medical Research Association, Leicester, UK; 4 Cardiovascular Analytics Group, PowerHealth Research Institute, Hong Kong SAR, P R China

**Keywords:** Finite element model, Spinal cord injury, Spinal cord stimulation, Axon model, Selectivity, Epidural electrical stimulation, Biomedical engineering, Biophysical models, Trauma

## Abstract

Epidural electrical stimulation (EES) enhances motor function recovery after spinal cord injury (SCI) by modulating distinct spinal pathways through dorsal epidural electrical stimulation (dEES) and ventral epidural electrical stimulation (vEES). The characteristics between dEES and vEES remain insufficiently explored. To address this, a rat spinal computational model was developed, integrating finite element analysis and nerve fiber modeling to simulate the effects of dEES and vEES. The potential distribution generated by EES was coupled with Aα-sensory and α-motor fibers to compute thresholds, saturation amplitudes, and selectivity indices across stimulation modes. The analysis showed that dEES exhibited lower thresholds and saturation amplitudes, while vEES achieved higher muscle selectivity. Multipolar stimulation dispersed currents across multiple spinal segments, reducing target muscle selectivity and increasing thresholds and saturation amplitudes compared to monopolar stimulation. Although stimulation frequency had little effect on selectivity in both dEES and vEES, higher frequencies in dEES reduced the stimulation intensity required to achieve maximum selectivity. These findings reveal key differences in activation characteristics between dEES and vEES and highlight their potential roles in neuromodulation. Most importantly, we provided a fiber-level explanation for these differences in rats and supplemented our findings with a comparative analysis of previous studies. These insights lay a foundation for future rodent experiments aimed at optimizing epidural stimulation strategies.

**Supplementary Information:**

The online version contains supplementary material available at 10.1038/s41598-025-19555-w.

## Introduction

Spinal cord injury (SCI) refers to damage to the spinal cord structure caused by trauma or disease, resulting in partial or complete loss of motor, sensory, and even autonomic functions^1–3^. Restoring walking ability is one of the primary goals of SCI rehabilitation, and achieving this objective largely depends on the precise activation and modulation of specific muscle groups to generate rhythmic gait patterns^4^. In recent years, EES, an invasive neuromodulation technique, has emerged as a promising approach in SCI rehabilitation because of its effectiveness in promoting gait recovery and enhancing muscle control^5,6^. The underlying mechanism of EES is thought to involve direct electrical stimulation of specific spinal neural circuits, compensating for impaired signal transmission from the brain to the spinal cord, and enabling neurons in the affected regions to re-engage in motor control^7^. Spinal stimulation has been shown to influence the connectivity of stimulated circuits and enhance spinal tract plasticity by promoting neuronal reactivation and the reconstruction of spinal neural networks. Additionally, it helps regulate muscle activity to prevent atrophy resulting from denervation, demonstrating significant clinical potential^7,8^. However, the mechanisms underlying the selective activation of neural fibers and muscles by EES require further elucidation, particularly regarding the differential effects of dorsal and ventral stimulation under various electrode configurations and stimulation parameters.

Epidural electrical stimulation can be categorized into dEES and vEES based on the anatomical location of stimulation. Anatomically, each spinal segment controls a corresponding part of the body through a pair of spinal nerve roots. The dorsal root contains sensory nerve fibers, with Aα-sensory fibers playing a key role in spinal circuits. These fibers enter the spinal cord via the dorsal root ganglion and transmit sensory information from the skin and other body regions, earning the dorsal root the designation of the “sensory root“^9^. In contrast, the ventral root is composed chiefly of α-motor fibers that originate in the spinal cord and extend to peripheral muscles to regulate contraction, thereby earning the designation “motor root”^9^.

Previous studies have shown that Aα-sensory fibers and α-motor fibers exhibit differences in functional properties and ion-channel expression, which may influence their responses to electrical stimulation^10,11^. The mechanisms of dEES and vEES differ. In dEES, electrical stimulation primarily targets large-diameter Aα-sensory fibers within the dorsal root. These fibers transmit excitatory signals to spinal interneurons via monosynaptic and polysynaptic pathways, which subsequently activate motor neurons in the ventral horn. This indirect excitation mechanism facilitates muscle recruitment, and the activation outcome is highly dependent on stimulation parameters and electrode configuration^12,13^. Additionally, dEES can enhance spinal network coordination and plasticity by modulating sensory input, potentially facilitating gait restoration^7^. In contrast, vEES directly activates motor neurons, leading to immediate modulation of muscle contraction^11–14^. As Sahrp et al. have demonstrated, dEES engages sensory afferents in the dorsal roots, which then synaptically drive motor neurons and ultimately activate muscle fibers—an indirect, multi-synaptic mechanism that preserves the natural intraspinal circuitry. By contrast, vEES bypasses these spinal circuits and directly depolarizes motor neuron axons in a non-synaptic manner, producing very rapid muscle responses. While this non-physiological recruitment order can accelerate muscle fatigue, interleaved stimulation across multiple sites has been demonstrated to partially mitigate these effects^12^.

Muscle modulation via epidural spinal cord stimulation have primarily focused on the dorsal side, while the ventral approach has received comparatively little attention. Sharpe et al. implanted electrodes at multiple locations along the cervical spinal cord in nonhuman primates and recorded electromyographic responses in the upper limb. Their findings indicated that the recruitment threshold for ventral epidural stimulation was higher than that for dorsal epidural stimulation^12^. Hogan et al. developed a wireless stimulation system with a ventral electrode array for epidural spinal stimulation in rats. They proposed that the selective targeting in a point-to-point configuration was likely achieved through direct activation of α-motor neurons^14^. These observations are consistent with Sharpe’s findings, both suggesting that ventral and dorsal epidural stimulation regulate muscle activity through distinct spinal circuitry activation mechanisms.

Computational models provide a faster and more precise alternative for optimizing electrode placement, size, and configuration during EES^15–17^. The mechanisms of muscle modulation via electrical stimulation may vary depending on the stimulation site. vEES is likely to directly activate α-motor fibers near the spinal ventral horn, whereas dEES primarily targets Aα-sensory fibers. On the other hand, histological images of the lumbosacral spinal cord in rats reveal that the ventral white matter is thicker than its dorsal counterpart^18^. Current applied from the ventral side undergoes greater attenuation, ultimately leading to a reduced extracellular potential. Additionally, the curvature of Aα-sensory fibers differs from that of α-motor fibers. These complexities can significantly impact threshold and muscle selectivity outcomes. Computational models provide a relatively accessible analytical approach to address these challenges. By simulating the effects of electrical stimulation on both the ventral and dorsal spinal cord, these models help researchers understand how different stimulation parameters influence the activation and conduction of distinct nerve fibers. This facilitates the optimization of stimulation parameters for precise muscle modulation.

In this study, we constructed a computational model of the rat lumbosacral segments and the motor neuron pools controlling the leg muscles, based on the anatomical map. The study focuses on two antagonistic muscle pairs: Vastus Medialis (VM) and Semitendinosus (ST), which effect knee extension and flexion, respectively, and Tibialis Anterior (TA) and Gastrocnemius (GC), which govern ankle dorsiflexion and plantar-flexion^19–22^. These muscles work in pairs to control the fine movements and balance of the rat’s hind limbs, coordinating their activity during gait, jumping, and standing^23^. Previous studies have employed stimulation frequencies between 10 and 100 Hz to promote gait recovery after spinal cord injury, with 50 Hz emerging as the most commonly used value because it reliably elicits robust, fatigue-resistant muscle activation while avoiding the diminished efficacy and increased metabolic demand often observed at higher frequencies^7,24–26^. We first analyzed the threshold, saturation, and selectivity index of target muscles under dEES and vEES at 50 Hz, comparing three electrode configurations—monopolar, bipolar, and tripolar. Then, we selected 22 electrode configurations with the highest selectivity at 50 Hz and examined their selectivity and required stimulation intensity at 50 Hz, and 100 Hz.

## Materials and methods

### Volume conductor model

**Fig. 1:**
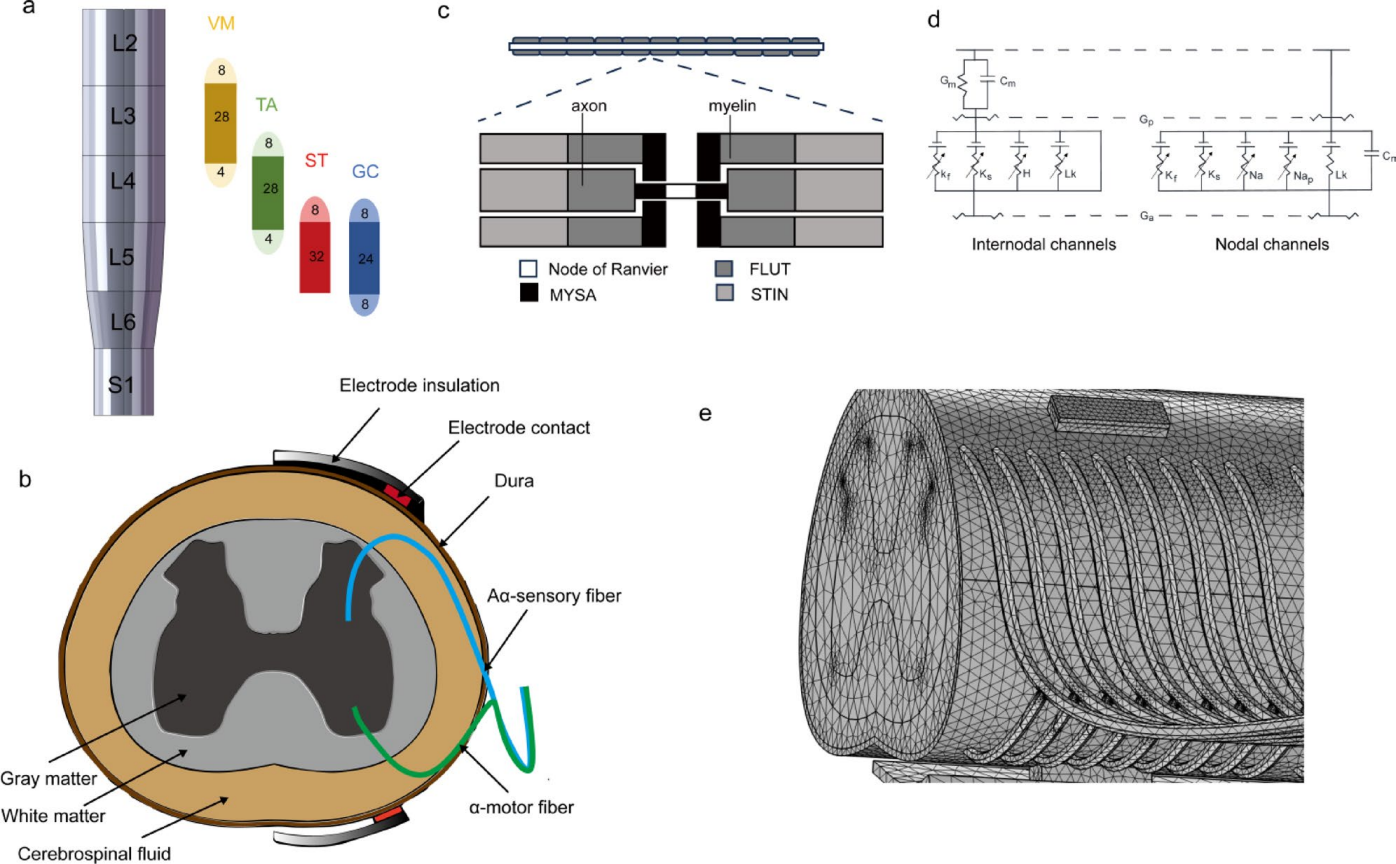
Spinal cord computational model. (**a**) Distribution of motor neuron pools for each muscle within the spinal cord: the motor neuron pool innervating the tibialis anterior (TA) is distributed across L3 (20%), L4 (70%), and L5 (10%); for the gastrocnemius (GC), it is located in L4 (20%), L5 (60%), and L6 (20%); for the vastus medialis (VM), the distribution is in L2 (20%), L3 (70%), and L4 (10%); and for the semitendinosus (ST), the pool spans L4 (20%) and L5 (80%). (**b**) The spinal cord segment at L4 includes gray matter, white matter, cerebrospinal fluid, dura, electrode contact, electrode insulation, Aα-sensory fibers, and α-motor fibers. (**c**) Multicompartment cable models of myelinated axons are constructed, with each node (NODE) represented by one segment and each internode divided into ten segments: two MYSA, two FLUT, and six STIN segments. (**d**) The node and internode segments are modeled with fast K⁺ (K_f_), slow K⁺ (K_s_), fast Na⁺ (Na), persistent Na⁺ (Na_p_), leak current (L_k_), and HCN (H) channels, along with nodal (C_n_), internodal (C_i_), and myelin (C_m_) capacitances, as well as axoplasmic (G_a_), periaxonal (G_p_), and myelin (G_m_) conductances. (**e**) The FEM mesh and the spatial arrangement of the fibers.

A three-dimensional model of the rat spinal cord lumbar-sacral segment L2-S1 was constructed based on anatomical maps and histological pictures^18^. The segment lengths of L2-S1 are 3.3, 2.8, 2.7, 2.8, 2.4, and 2.7 mm, respectively^27^ (Fig. 1a,b).

The spinal cord model consists of 10 different parts: gray matter, white matter, dorsal and ventral roots, cerebrospinal fluid, dura mater, epidural fat, vertebrae, peri-vertebral layer, electrode contact points, and electrode insulation. Representative cross-sectional planes of gray matter, white matter, dura mater, and epidural fat were first established for each segment.

These two-dimensional closed curves were processed using SolidWorks (v. 2022, Dassault Systèmes Deutschland GmbH, Stuttgart, Germany) to form three-dimensional solids. Finally, spinal roots and fibers with specific curvature trajectories were inserted. In the spinal roots, Aα-sensory fibers and α-motor fibers are arranged at regular intervals in the form of rootlets (Fig. 1e). Electrodes were placed at the center of each spinal segment with dimensions of 1000 μm by 300 μm. In monopolar mode, the distance between the active and return electrodes is very large, with 5 electrode configurations depending on electrode placement (Fig. S1a). In bipolar mode, the active and return electrodes are placed relatively close together, with 10 electrode configurations (Fig. S1b). In tripolar mode, the active electrode is in the middle and the return electrodes are at both ends, with 10 electrode configurations (Fig. S1c). Therefore, dEES and vEES each have 25 electrode configurations across the three stimulation modes. Detailed electrode configurations are provided in the supplementary material (Fig. S1).

### Physics

We used the commercial software COMSOL (v.6.0, COMSOL Inc., USA) to process the established three-dimensional model and assign conductivity values to each tissue^28^. Detailed tissue parameters are provided in Table 1. We used methods from previous studies to generate curve coordinates for WM and spinal roots in COMSOL and oriented.their anisotropic conductivity tensors^29^. The curve coordinate system employs a diffusion method, which supports the handling of cross-sectional variations and sharp bends, aligning with the curved trajectories of the fibers. The inlet is positioned on the upper surface of the rostral-side WM, the outlet is set on the terminal surface of each root and the lower surface of the caudal-side WM, and the wall boundary is defined on the remaining lateral surfaces.

**Table 1:** Tissues’ conductivities considered in the finite element model^13^.

Tissue	Conductivity (S/m)
Gray matter	0.23
White matter (longitudinal)	0.6
White matter (transverse)	0.083
Cerebrospinal fluid	1.7
Dura	0.6
Epidural fat	0.04
Vertebral bone	0.02
Saline	2.0

The quasi-static approximation was used to calculate the electric potential distribution within the volume conductor during electrical stimulation, simplifying Maxwell’s equations to the Laplace Eq. (2)^9,30^.

1 $$\:\overrightarrow{\nabla\:}\:\cdot\:\overrightarrow{J}\:\left(x\right)=0\:\forall\:x\in\:{\Omega\:}$$

Where$\:\:\overrightarrow{J}\:\left(x\right)=\sigma\:\left(x\right)\overrightarrow{E}\left(x\right)$ represents the current density, $\:\overrightarrow{E}\left(x\right)=-\overrightarrow{\nabla\:}V\left(x\right)$ is the electric field derived from the electric potential, $\:\sigma\:\left(x\right)$ is the conductivity tensor at any point $x~ \in \Omega$, interior of the volume conductor^30^. The boundary condition at the electrode contact surface is defined as a boundary current source, with an injected current density of $\:{Q}_{j}\:=\:1A/{m}^{2}$. This value is selected solely to satisfy the finite-element solver and is unrelated to the stimulus parameters used in in-vivo experiments on rats. Under the quasi-static assumption, reciprocity ensures that all results scale linearly with stimulus intensity, obviating the need for additional simulations^31,32^.

2 $$\:\left(\overrightarrow{{J}_{1}}\left(x\right)-\overrightarrow{{J}_{2}}\left(x\right)\right)\bullet\:\overrightarrow{{n}_{2}}=\frac{{Q}_{j}}{{A}_{S}}\:\forall\:x\in\:S\:\:$$

Where S is the boundary where the condition is applied, $\:{A}_{S}$ its area, and $\:\overrightarrow{{J}_{1}}$, $\:\overrightarrow{{J}_{2}}$,and $\:\overrightarrow{{n}_{2}}$ represent the current densities on each side of the boundary and the normal vector to the boundary, respectively^30^.

The outermost boundary of the model was assigned a Dirichlet condition (V = 0), assuming no current flow across the external surface. After defining the material properties and boundary conditions, a free tetrahedral mesh was generated (Fig. 1e). The maximum element size was set to 600 μm and the minimum element size to 2 μm. In COMSOL, the maximum element size caps each mesh edge: lowering it refines geometry and field detail, while raising it speeds the solve at some loss of accuracy. The minimum element size sets a floor, blocking tiny elements that would unduly prolong computation.

### Axon model

Axon activation thresholds are lower than those of cell somas and dendrites, and the proportion of axonal fibers in the spinal cord is much higher than that of cell somas and dendrites. Therefore, in the simulation, we only established axon models in the spinal cord, all of which were built in Neuron (v. 8.2) and Python (v. 3.9.8) (Fig. 1c,d)^32^.

Previous studies have shown that epidural stimulation in the spinal cord primarily affects large primary afferent fibers in the dorsal roots and efferent fibers in the ventral roots. Even with strong stimulation, EES cannot activate the cell somas and dendrites of interneurons and motor neurons^13,33–35^. Due to differences in membrane capacitance and resistance, electrical stimulation primarily activates larger myelinated fibers, following an inverse recruitment order^13,36^. As a result, our simulations focus exclusively on Aα fibers and do not account for myelinated Aβ fibers or unmyelinated C fibers. Additionally, the cell somas of interneurons and motor neurons located in the spinal cord are not considered in the calculations. Based on previous measurements, the diameters of Aα-sensory fibers in the dorsal root and α-motor fibers in the ventral root are both 9 µm^37^. Studies have shown physiological differences between sensory and motor fibers in the spinal cord; sensory fibers of the same diameter have a lower threshold than motor fibers^11^. To reflect this difference in the computational model and make the simulation more consistent with experimental conditions, we used Gaines modified α-motor and Aα-sensory fibers based on the MRG model. Both types of nerve fibers have the same structure, consisting of nodes of Ranvier and internodes. The MRG model is a mammalian myelinated-axon “double‐cable” framework developed by McIntyre and Grill’s group through the incorporation of ion‐channel dynamics. It subdivides each myelinated region between adjacent Ranvier nodes into ten compartments: two Myelin‐Attachment Segments (MYSA), two Paranodal Main Segments (FLUT), and six Internodal Segments (STIN)^10,38^.The node and internode segments are modeled with fast K⁺ (K_f_), slow K⁺ (K_s_), fast Na⁺ (Na), persistent Na⁺ (Na_p_), leak current (L_k_), and HCN (H) channels, along with nodal (C_n_), internodal (C_i_), and myelin (C_m_) capacitances, as well as axoplasmic (G_a_), periaxonal (G_p_), and myelin (G_m_) conductances^10,38^. (Fig. 1c). The main modification in the Gaines model is the addition of a fast K^+^ channel at the nodes and the inclusion of multiple channels in the internodes, more accurately reflecting the differences between motor and sensory fibers (Fig. 1d).

Based on anatomical data, a pool of 40 motor neurons was established for each muscle in the spinal cord segments^39,40^. The motor neuron pool for the Tibialis Anterior (TA) is distributed at L3 (20%), L4 (70%), and L5 (10%); for the Gastrocnemius (GC), it is distributed at L4 (20%), L5 (60%), and L6 (20%); for the Vastus Medialis (VM), it is distributed at L2 (20%), L3 (70%), and L4 (10%); and for the Semitendinosus (ST), it is distributed at L4 (20%) and L5 (80%) (Fig. 1a). The corresponding numbers of Aα-sensory fibers and α-motor fibers were also established accordingly.

### Evaluating axonal response to stimulation

The voltage distribution exported from COMSOL is used as the input source for extracellular stimulation of the axon models to calculate changes in the axonal membrane potential. Python is used to insert the voltage distribution into the corresponding sections of the axon, and the extracellular mechanism built into Neuron is used to process the imported voltage^32^. The stimulation waveform is a symmetric biphasic waveform with a pulse width of 200 µs. The stimulation intensity is gradually increased to determine the activation threshold of the axons. The current intensity ranges dynamically from 25 to 1000 µA, with a step size of 25 µA. For each fiber, when the action potential successfully propagates from the initial node of Ranvier to the terminal node, the fiber is considered recruited. Threshold and saturation were defined, for each target muscle, as the current amplitudes required to recruit 10% and 90% of the total population of nerve fibers innervating that muscle, respectively^13^.

### Selectivity evaluation

Due to the distinct positional distributions of dorsal root sensory fibers and ventral root motor fibers, dEES and vEES engage different spinal pathways. dEES tends to activate dorsal root sensory fibers, while vEES more easily activates ventral root motor fibers^12,13,26^. To assess muscle selectivity in EES recruitment, the Selectivity Index (SI) is calculated using the following formula:

3 $$\:{SI}_{M}^{E}=\:\underset{{amp}\in\:{DR}}{{max}}\left\{{R}_{M}^{E}\left(amp\right)-\frac{1}{{N}_{musc}-1}{\sum\:}_{n\ne\:m}^{N}{R}_{n}^{E}\left(amp\right)\right\}\:\:$$

where E represents a specific electrode configuration, M represents the target muscle, DR represents the dynamic range of stimulus amplitude, R is the recruitment level of the muscles, and N is the total number of muscles. The range of SI is from − 1 to 1, where a value of 0 indicates that the average activation rate of the target muscle is the same as that of other muscles.

### Statistical analysis

**Fig. 2:**
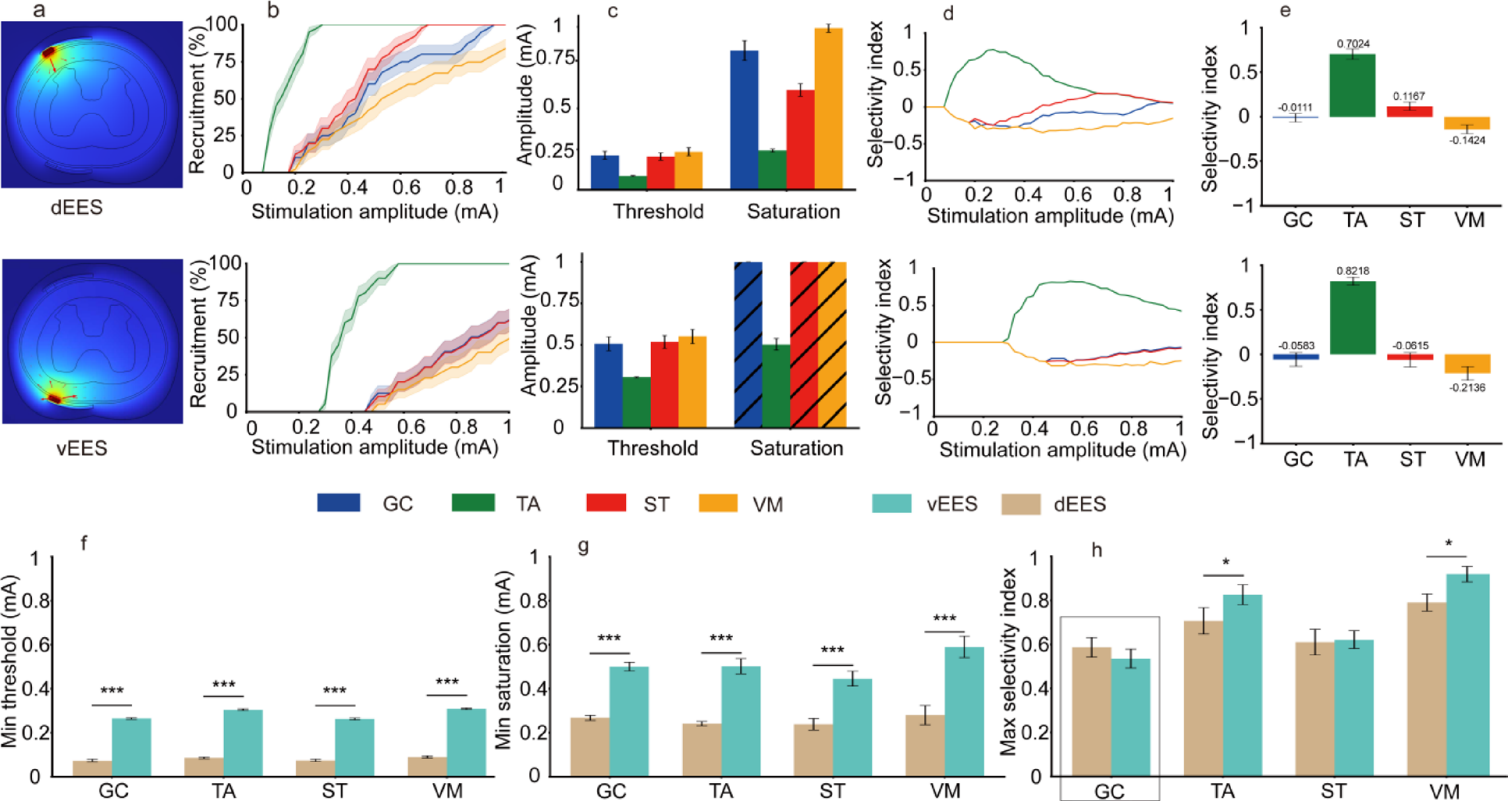
Computational modeling results comparing dEES and vEES under monopolar stimulation mode. (**a**) Electric potential distribution in the L4 spinal segment for dEES (top) and vEES (bottom). (**b**) Recruitment curves of target muscle fibers under dEES (top) and vEES (bottom). The shaded region indicates the standard deviation of the total-fiber recruitment rate at each stimulation intensity. (**c**) Threshold (10% activation) and saturation (90% activation) amplitudes for individual muscles. Values exceeding 1 mA are shaded. (**d**) Selectivity index variation with stimulation intensity. (**e**) The selectivity index from (d) was chosen as the maximum value within this electrode configuration, falling between the threshold and saturations. (**f**–**h**) Statistical comparisons of minimum thresholds (f), minimum saturations (g), and maximum selectivity indices (h) between dEES and vEES. Black boxes highlight cases where dEES outperforms vEES. Data are reported as mean ± SD (a one-sample one-sided t test for bootstrapped samples, *n* = 10,000, *p < 0.05, ***p < 0.001).

**Fig. 3:**
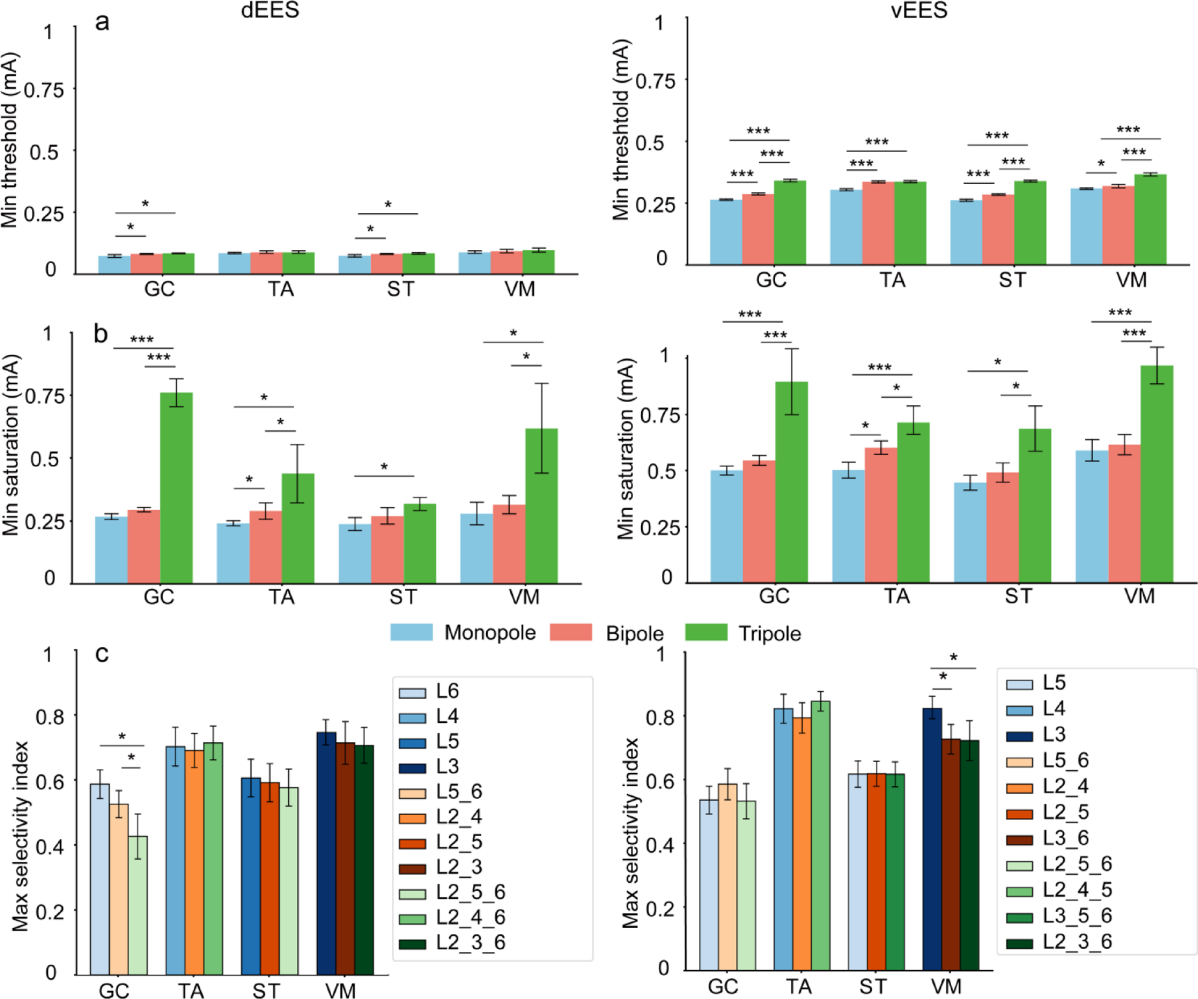
Comparison of stimulation modes in the computational model. (**a**–**c**) Statistical comparisons of minimum thresholds (a), minimum saturations (b), and maximum selectivity indices (c) between three stimulation modes (blue for monopole, red for bipole, and green for tripole), with dEES on the left and vEES on the right. Data are reported as mean ± SD Data are reported as mean ± SD (one-way ANOVA with Bonferroni correction for bootstrapped samples, *n* = 10,000, *p < 0.05, ***p < 0.001).

**Fig. 4:**
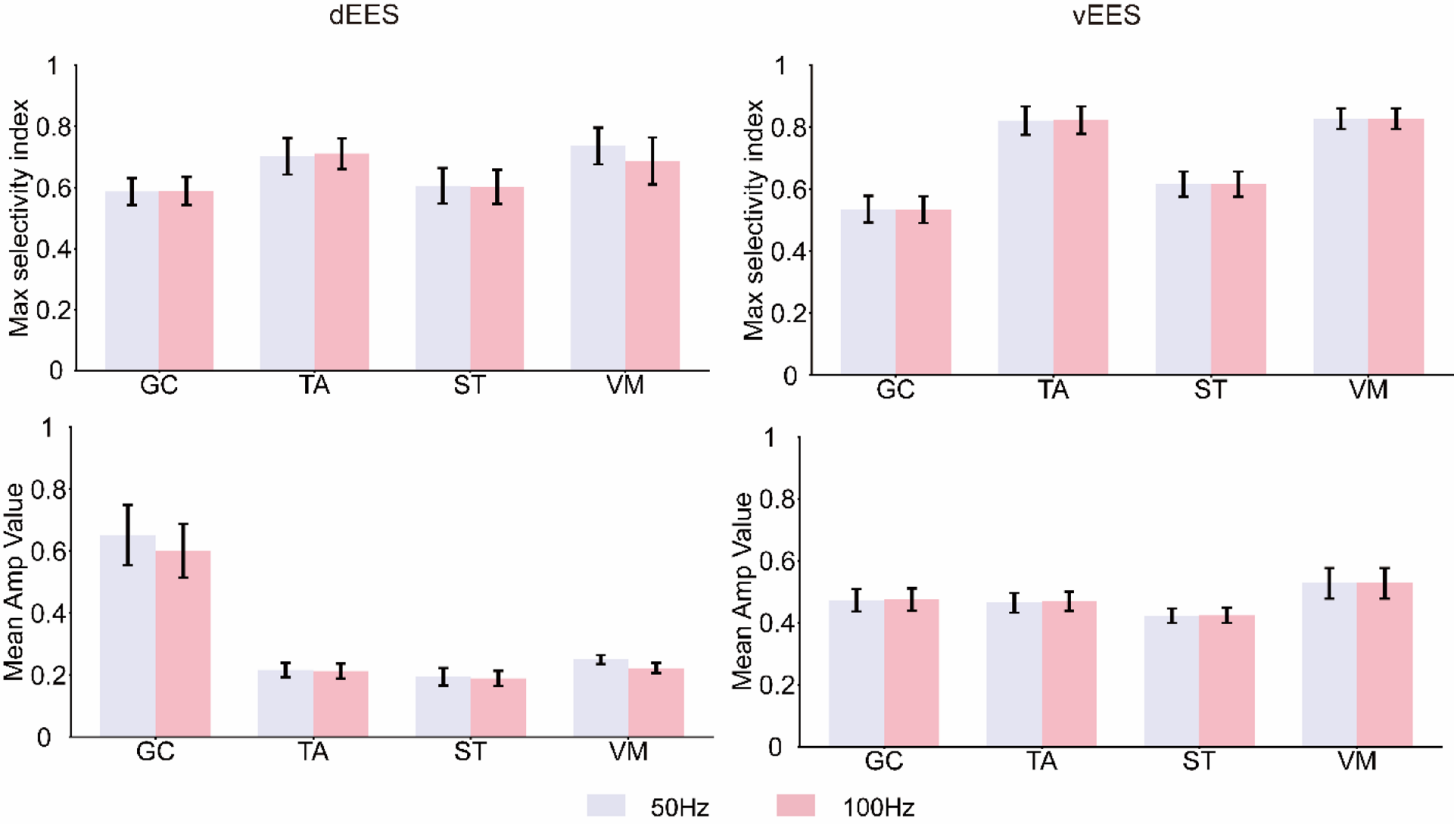
Effects of frequency on the maximum selectivity index and amplitude values under the monopolar stimulation mode. (**a**) Maximum selectivity indices (top) for each muscle under dEES at 50 Hz, and 100 Hz. Stimulation intensity (bottom) required to achieve the maximum selectivity index at each frequency. (**b**) Same as the (a) for vEES. Data are reported as mean ± SD (a one-sample one-sided t test for bootstrapped samples, *n* = 10,000, *p < 0.05, ***p < 0.001).

The sampled fibers undergo bootstrap analysis (*n* = 10,000), i.e., each target muscle is innervated by 40 modeled fibers. After exporting the original set of *n* = 40 fiber responses from COMSOL, we used Python to draw a new sample of m = 40 fibers with replacement—meaning that any individual fiber could be selected multiple times or not at all in a given draw. We repeated this procedure 10,000 times, producing K = 10,000 bootstrap data sets. For each bootstrap replicate, we computed the muscle’s activation threshold, saturation current, and selectivity index, and then calculated the mean and standard deviation across the K replicates for subsequent statistical testing. Recruitment curves for each muscle were plotted, and the selectivity index was calculated^26^. For result comparison, we identified the lowest values across all electrode configurations within each stimulation mode, defining them as the Minimum Threshold and Minimum Saturation (Figs. 2f,g,  3a,b). We selected the highest value across all configurations within each mode, termed the Maximum Selectivity Index (Figs. 2h, 3c, 4a,b). The detailed selection process is presented in the supplementary materials (Fig. S2). All data are reported as mean ± standard deviation unless specified otherwise, statistics obtained by Bootstrapping, a one-sample one-sided t test or one-way ANOVA with Bonferroni correction for Bootstrapped samples, *n* = 10,000, * *p* < 0.05, *** *p* < 0.001.

## Results

### Comparison of threshold, saturation, and selectivity between dEES and vEES

To systematically evaluate the efficacy of dorsal and ventral epidural electrical stimulation (dEES and vEES) in the L4 spinal segment, we analyzed three key parameters: threshold, saturation, and selectivity index. The electric potential distribution generated by both stimulation modalities was simulated using a finite element model (Fig. 2a), and axon models were coupled to quantify the muscle recruitment. The recruitment curve was plotted (Fig. 2b), and the threshold and saturation of the target muscle were determined (Fig. 2c). The variation in the selectivity index with stimulation intensity (Fig. 2d), and the maximum value within the given electrode configuration was identified (Fig. 2e). For comparative analysis, the minimum threshold and saturation across all electrode configurations within each stimulation mode (monopole, bipole, tripole) were selected, and the maximum selectivity index was extracted (see Methods and Fig. S2 for selection criteria). Results for the monopolar mode are presented below; data for other modes are provided in Supplementary Figs. S3, S4.

dEES exhibited significantly lower thresholds compared to vEES across all target muscles (Fig. 2f). Specifically, the minimum thresholds for dEES were ~ 71–72% lower than those for vEES (GC: 0.073 ± 0.006 mA vs. 0.264 ± 0.003 mA; TA: 0.085 ± 0.003 mA vs. 0.304 ± 0.004 mA; ST: 0.074 ± 0.005 mA vs. 0.262 ± 0.004 mA; VM: 0.089 ± 0.005 mA vs. 0.309 ± 0.003 mA; all *p*<0.001). A similar trend was observed for saturation (Fig. 2g), with dEES requiring 47–52% lower currents than vEES (GC: 0.267 ± 0.011 mA vs. 0.500 ± 0.020 mA; TA: 0.241 ± 0.010 mA vs. 0.502 ± 0.035 mA; ST: 0.238 ± 0.026 mA vs. 0.446 ± 0.033 mA; VM: 0.270 ± 0.028 mA vs. 0.561 ± 0.006 mA; all *p*<0.001).

The maximum selectivity indices revealed a muscle-specific preference (Fig. 2h). While dEES exhibited a nominally higher selectivity for the GC (0.5869 ± 0.0442 vs. 0.5354 ± 0.0433), this difference did not reach statistical significance (*p* = 0.1231). In contrast, vEES demonstrated significantly higher selectivity for the TA (0.8218 ± 0.0453 vs. 0.7024 ± 0.0596, *p* < 0.05) and VM (0.8297 ± 0.0332 vs. 0.7362 ± 0.0597, *p* < 0.05). No significant difference was observed for the ST (0.6057 ± 0.0581 vs. 0.6169 ± 0.0411, *p* = 0.4125).

### Comparison of thresholds, saturations, and selectivity between stimulation modes

The minimum threshold, minimum saturation, and maximum selectivity index were compared across monopole, bipole, and tripole (Fig. 3a–c). Details on the selection process are in Supplementary Fig. S2.

For the minimum threshold, no significant differences were observed in TA and VM across the three stimulation modes in dEES. However, GC and ST exhibited significantly lower thresholds under the monopole (Fig. 3a, left). In vEES, all four muscles showed significantly lower thresholds under monopole compared to bipole and tripole, with tripole exhibiting the highest threshold (Fig. 3a, right).

For the minimum saturation, in dEES, all four muscles exhibited significantly lower saturation under monopole compared to tripole. Additionally, TA had significantly lower saturation under monopole than bipole, while GC, TA, and VM showed significantly lower saturation under bipole compared to tripole (Fig. 3b, left). In vEES, the saturation of all four muscles were significantly lower under monopole than tripole, and those under bipole were also significantly lower than tripole. Moreover, TA had significantly lower saturation under monopole compared to bipole (Fig. 3b, right).

For the maximum selectivity index, in dEES, the tripole for GC was significantly lower than both bipole and monopole (Fig. 3c, left). In vEES, the monopole for VM was significantly higher than both bipole and tripole (Fig. 3c, right).

### Effect of frequency on selectivity and required stimulation intensity

We analyzed 22 electrode configurations (Fig. 3c) to compare the effects of 50 Hz, and 100 Hz on the maximum selectivity index and the required stimulation intensity (Fig. 4). For clarity, only monopole data are shown, while bipole and tripole results are in Supplementary Figs. S5 and S6.

In dEES, the maximum selectivity index varies little across frequencies (Fig. 4a, top), while the required stimulation intensity decreases as frequency increases (Fig. 4a, bottom), though not significantly.

In vEES, both metrics remain largely unchanged (Fig. 4b).

## Discussion

We developed a 3D computational model of the rat spinal cord using an existing axon model to compare muscle thresholds, saturations, and selectivity across dEES, vEES, and different stimulation modes. Results showed that dEES required lower thresholds and saturations, while vEES provided stronger selectivity, highlighting differences in neural regulation and muscle activation. monopolar stimulation enhanced selectivity and required lower intensity, whereas multipolar stimulation activated muscles more broadly but at higher intensity.

Our simulation results showed that in vEES, the minimum thresholds and minimum saturations were significantly higher than in dEES, which may be attributed to several factors: (i) Physiological differences in nerve fibers, particularly ion channel variations. The Gaines-modified MRG model used in this study shows that Aα-sensory fibers have significantly lower thresholds than α-motor fibers of the same diameter^11,41^. (ii) The ventral spinal cord has a thicker tissue medium than the dorsal side^18^leading to greater current attenuation. As a result, α-motor fibers receive lower extracellular potential, requiring higher stimulation amplitudes for activation. Differences in fiber curvature also influence activation.

vEES generally achieves a higher maximum selectivity index than dEES, likely due to fiber properties. α-motor fibers are less sensitive to electrical stimulation than Aα-sensory fibers and harder to activate. When the electrode is placed near the motor pool of target muscle, vEES more effectively activates α-motor fibers while sparing non-target muscles. In contrast, dEES more easily excites Aα-sensory fibers, leading to activation of motor neuron pools in nearby non-target muscles (Fig. 2b,d,e).

An exception occurs in monopolar mode, where GC selectivity is lower in vEES (0.53536) than in dEES (0.58695) (Figs. 2h, 3c). This is due to different stimulation sites: ventral stimulation at L5 of monopole and dorsal stimulation at L6 of monopole (Figs. 2h, 3c). In L5 of monopole, the proximity of GC and ST motor neuron pools causes unintended ST activation, reducing GC selectivity. This suggests that placing electrodes in densely packed motor neuron areas does not always enhance selectivity. Instead, targeting regions with fewer neuron pools may improve selectivity, though at the cost of higher stimulation intensity (Fig. 4a,b, bottom). Future studies should optimize stimulation parameters to balance selectivity and current requirements, further clarifying the neural regulation differences between dEES and vEES.

The high efficiency of dorsal stimulation and the strong selectivity of ventral stimulation suggest they could complement each other for different targets. Future research should explore combined dorsal-ventral stimulation, integrating electrode optimization and parameter tuning. By leveraging dorsal indirect activation of monosynaptic circuits and ventral direct activation of motor fibers, this approach could improve spinal pathway assessment advancing spinal cord stimulation for neural regulation.

Bipole and tripole require slightly higher thresholds than monopole but result in significantly increased saturation levels due to current dispersion across multiple spinal segments, leading to widespread muscle activation (Fig. 3a,b). In dEES, monopole shows the highest selectivity for GC, followed by bipole, with tripole being the lowest (Fig. 3c, left). In vEES, monopole achieves the highest selectivity for VM compared to bipole and tripole (Fig. 3c, right). Monopolar stimulation generally provides higher selectivity, while multipolar stimulation offers more balanced activation, making it better suited for multi-muscle coordination, such as gait recovery and movement control.

Another finding is that in both dEES and vEES, the tripolar mode with dispersed electrode configurations (spanning multiple spinal segments) achieves a higher selectivity index. For example, in dEES, the optimal configurations for GC, TA, ST, and VM are L2_5_6, L2_4_6, L2_5_6, and L2_3_6, respectively (Fig. 3c). These electrode placements are relatively dispersed rather than concentrated in the motor neuron pools of the target muscles. This is because segments near the target contain many non-target muscle motor neurons, and placing electrodes too closely together can lead to excessive current concentration, activating unwanted muscles.

Therefore, when designing multipolar configurations, active electrodes should be positioned at the center of the target muscle’s motor neuron pool, while return electrodes should be placed away from non-target motor neuron pools. This highlights the broad muscle activation effect of multipolar stimulation. Future designs should incorporate high-resolution electrode arrays to enhance selective muscle activation through precise electric field shaping^42,43^.

In vEES, both GC and ST achieve their highest selectivity index with the same monopolar configuration, L5 of monopole, while in dEES, it occurs with L2_5_6 of tripole(Fig. 3c). This may be due to the proximity of their motor neuron pools within the spinal cord, leading to similar selectivity effects. This finding suggests the potential for coordinated regulation of related muscles.

For most muscles except GC, the current required to reach maximum selectivity is lowest in monopolar stimulation, followed by bipolar, and highest in tripolar stimulation (Figs. 4, S5, S6). Therefore, the choice of stimulation mode should be based on specific goals, such as enhancing sensory input, improving gait coordination, or strengthening target muscles, with stimulation parameters optimized accordingly.

We also compared the effects of different frequencies on selectivity, focusing on the 10–100 Hz range commonly used for gait recovery, as excessively high frequencies can cause sensory abnormalities and muscle fatigue^24,25^. Using 22 electrode configurations with the highest selectivity at 50 Hz, we analyzed selectivity and stimulation intensity at 50 Hz, and 100 Hz. In dEES, increasing frequency had little effect on the maximum selectivity index, while the current required for peak selectivity slightly decreased (Fig. 4a). In vEES, both selectivity and required stimulation intensity remained largely unchanged (Fig. 4b). The differing responses in dEES and vEES may be due to fiber properties, as α-motor fibers are harder to activate than Aα-sensory fibers, making muscle responses in vEES less sensitive to current changes^11,41^.

This study utilized the Gaines-modified MRG model, enabling a direct comparison of muscle modulation differences between dEES and vEES stimulation^10^. In our computational model, the simulated muscle activation thresholds for dEES and vEES, as well as their numerical differences, were largely consistent with previous experimental studies. Capogrosso et al. modeled and experimentally examined dorsal epidural electrical stimulation in rats, reporting a monopolar stimulation threshold range of 100–150 µA for facilitating locomotion^13^. Our model estimated the minimum threshold to be 73–89 µA. For ventral stimulation, Hogan et al. stimulated the ventral cervical spinal segments in rats and reported an average threshold of 314.5 ± 21.9 µA across all monopolar modes^14^. Our model estimated a threshold range of 262–309 µA. The lower threshold in our simulation may be attributed to the electrode placement closer to the spinal root, known as lateral stimulation, where nerve fibers are exposed to denser current, facilitating activation at lower thresholds. Additionally, our model extends the work of Hogan et al. by applying stimulation to the same spinal segments dorsally and ventrally to explore differences in muscle recruitment. Sharp et al. compared multiple electrical stimulation methods in the cervical spinal cord of nonhuman primates, reporting average thresholds of 187 ± 25 µA for dorsal epidural stimulation and 278 ± 50 µA for ventral epidural stimulation^12^. These differences were also reflected in our computational model. Additionally, Sharp et al.‘s analysis of flexor and extensor muscle groups showed that vEES had a higher selectivity index (≈ 0.58) than dEES (≈ 0.45)^12^. Similarly, Badi et al. examined peripheral nerve stimulation and found no significant difference in selectivity index between 100 Hz and 50 Hz stimulation frequencies, a result consistent with our study^44^. Aligning with our simulation results, Thus, our simulation results are consistent with previous experimental studies, supporting the validity of our model.

There are certain limitations in our computational model, such as the model being based on past anatomical maps and histological pictures rather than more precise MRI or CT scans. To facilitate the finite element model solution, some simplifications were made in the three-dimensional entities, such as modeling the vertebrae and epidural fat as more regular solids. The electrode position in this study is on the spinal cord epidural. Compared to percutaneous electrical stimulation where electrodes are placed on the body, the impact of simplified geometry in this study is negligible and within an acceptable range. However, Zander’s study indicates that the position of the electrode relative to the vertebrae in EES can affect the fiber threshold^45^. This was not considered in the present study. In the future, models could be constructed using MRI and CT scans to increase the complexity of the model and achieve more accurate simulation results^4,46^. Previous studies report spinal dura conductivities ranging from 0.02 to 0.6 S/m^47,48^. To align our model with the boundary conditions used in related simulations, we adopted the upper bound (0.6 S/m). This assumption may account for the remaining gaps between our simulations and experimental observations. Future work should systematically probe how different dura conductivities affect activation amplitude and selectivity, and validate the findings in animal experiments to identify the conductivity value that best reflects physiological reality. Another principal limitation of this study is the absence of new in-vivo animal data to validate the simulation findings. Although we benchmarked the model against previously published experimental results, direct experimental replication remains essential for establishing causal validity. In addition, we employing a single fiber diameter may influence the precise evaluation of muscle activation thresholds and saturation values, a choice that facilitated mechanistic comparisons between dEES and vEES. Future work will therefore pursue two complementary directions: (i) empirically corroborating the present simulations through dedicated animal experiments and (ii) extend the model to incorporate realistic diameter distributions and ion-channel heterogeneity, enabling a systematic exploration of how muscle recruitment interacts with the stimulation paradigm.

The complementary selectivity and activation efficiency of vEES and dEES offer insights for coordinated gait regulation, enabling and precise neuromodulation. Multipolar stimulation provides balanced selectivity across multiple muscles and synchronizes activation, making it ideal for complex gait coordination. Future research should focus on closed-loop control algorithms that dynamically adjust electrode configurations based on gait phases for precise muscle activation. Miniaturized electrode arrays can achieve precise nerve fiber targeting, while adaptable multi-electrode systems can generate complex electric fields for selective muscle regulation. Advanced electrode technologies with switchable polarity allow for optimized stimulation at different rehabilitation stages, supporting diverse muscle training needs.

## Conclusion

In this study, we revealed the differences in muscle threshold, saturation, and selectivity between dEES and vEES through a computational model. These differences suggest that the variation in neuromodulation mechanisms and muscle activation effects between dEES and vEES stem from the differing characteristics of sensory and motor fibers. We also identified notable variations in activation outcomes across different stimulation modes. Additionally, in dEES, increasing frequency had little effect on the maximum selectivity index, while the current required for peak selectivity slightly decreased. In vEES, both selectivity and required stimulation intensity remained largely unchanged. Together, We provided a fiber-level explanation for these findings in rats and further supported our conclusions through comparative analysis with previous studies. These insights lay a foundation for future rodent experiments aimed at optimizing epidural stimulation strategies.

## Supplementary Information

Below is the link to the electronic supplementary material.

Supplementary Material 1

Supplementary Material 2

## Data Availability

The datasets used can be found at https://github.com/Yim1568/compare_D_V.
