# Supplementary material for: Comparison of activation and selectivity in dorsal and ventral epidural spinal cord stimulation in rats: a computational modeling study
Source: Sci Rep. 2025 Oct 13;15:35711. doi: 10.1038/s41598-025-19555-w (PMC12518515; doi:10.1038/s41598-025-19555-w)
Supplement: Supplementary file 1 — Supplementary Material 1 [file 41598_2025_19555_MOESM1_ESM.docx]

**Supplementary Materials for**

**Comparison of Activation and Selectivity in Dorsal and Ventral Epidural Spinal Cord Stimulation in Rats: A Computational Modeling Study**

**
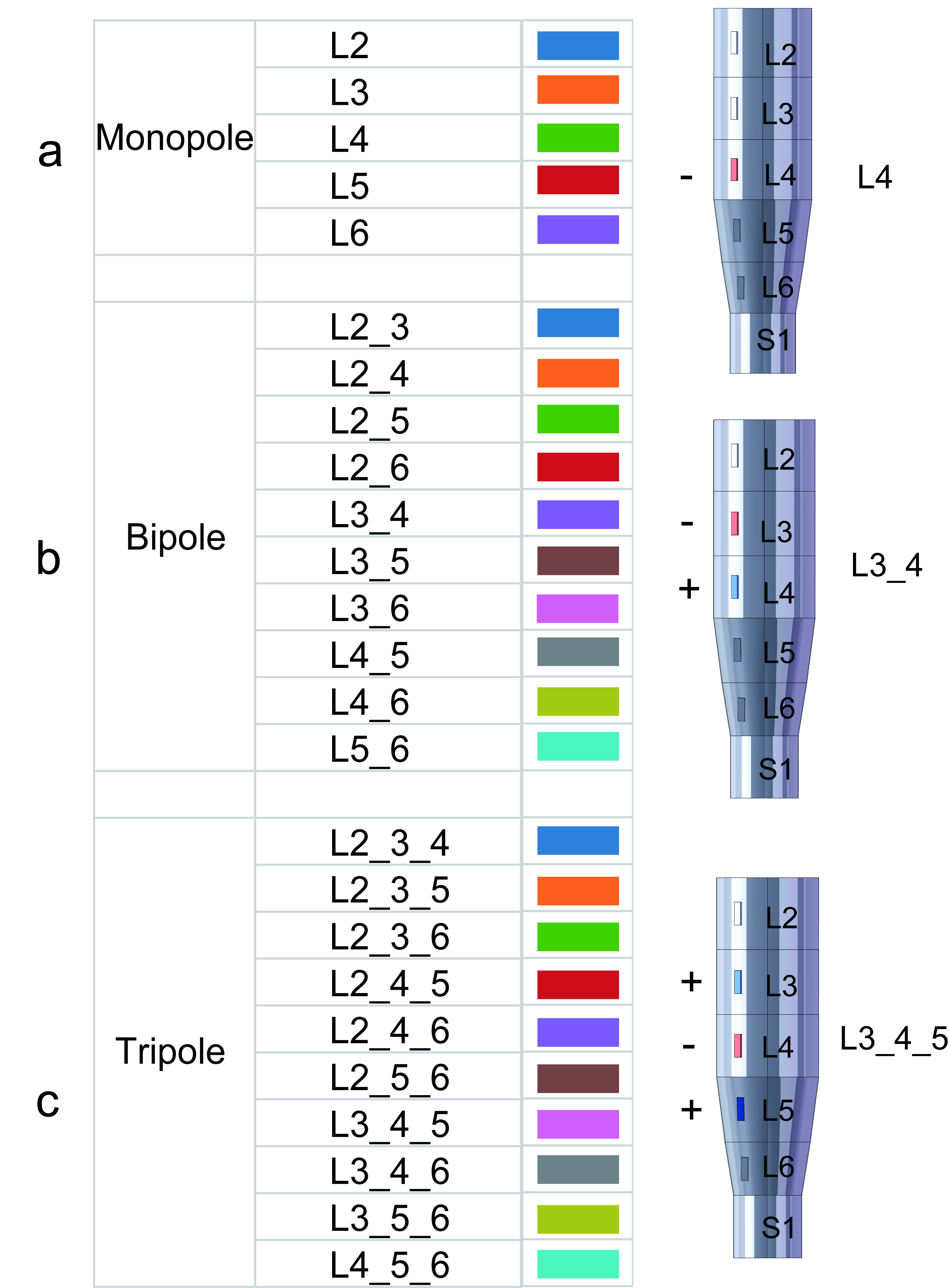
**

**Figure S1.** Electrode configurations in the three stimulation modes for dEES. (**a**) Monopolar stimulation mode, with a total of 5 electrode configurations, demonstrated using L4 as an example. The letter L represents the lumbar spinal segment where the electrode is placed. In each of the three stimulation modes, every electrode configuration is represented by a unique color, which corresponds to the colors shown in (Figure S2(a), (b)). Red represents the cathode, while blue represents the anode. (**b**) Bipolar stimulation mode, with a total of 10 electrode configurations, demonstrated using L3_4 as an example. (**c**) Tripolar stimulation mode, with a total of 10 electrode configurations, demonstrated using L3_4_5 as an example. The configurations for vEES are the same as those above.

**

**

**Figure S2.** Schematic diagram of the selection and comparison of the maximum selectivity index. (**a**) In the calculation of the selectivity index for the three stimulation modes in dEES, each color represents a specific electrode configuration, which can be found in Figure S1. (**b**) Same as (a), but for vEES. (**c**) Selection of the maximum value and corresponding electrode configuration for each mode from (a) and (b). Blue represents vEES, red represents dEES, The three color gradients represent the three different stimulation modes (**d**) Comparison of dEES and vEES across the three stimulation modes: monopole, bipole, and tripole. Brown represents dEES, cyan represents vEES. (**e**) Comparison of the three stimulation modes within dEES and vEES, respectively. The color scheme represents different stimulation modes: blue for monopole, red for bipole, and green for tripole. Data are reported as mean ± SD (a t-test or one-way ANOVA with Bonferroni correction for bootstrapped samples, n=10,000, *p < 0.05, ***p < 0.001).

**
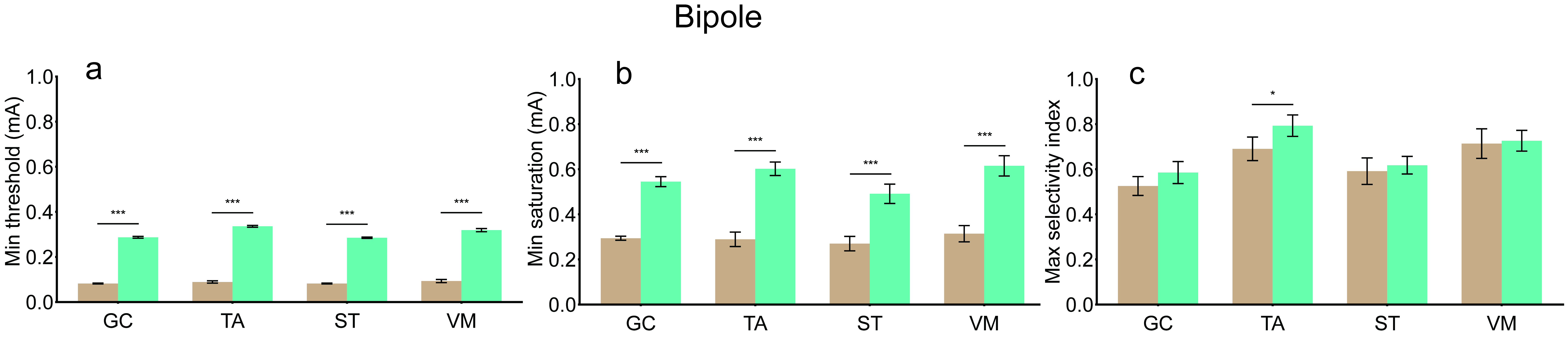
**


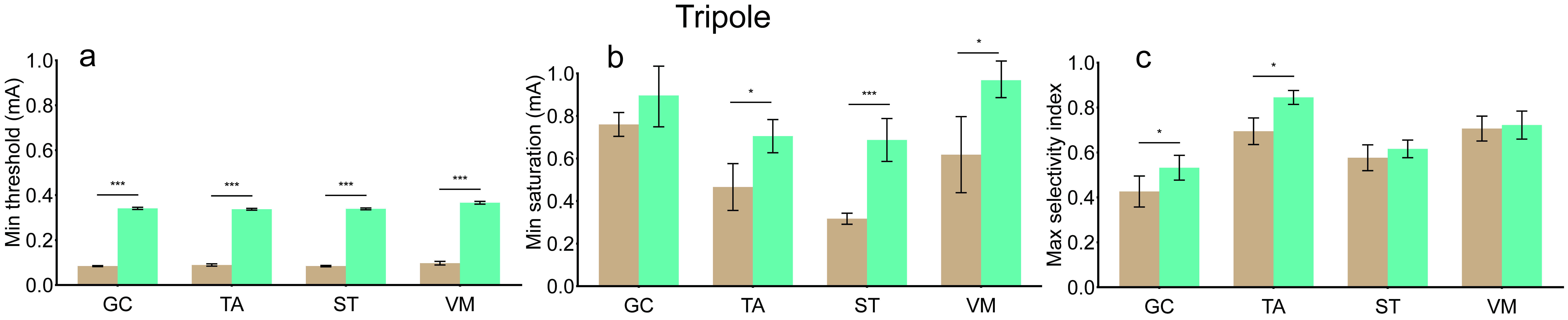
**Figure S3.** Simulation results of dEES and vEES in the computational model under bipolar stimulation mode. (**a**) Comparison of the minimum thresholds between dEES and vEES. The thresholds shown are the minimum values across all electrode configuration in bipole mode. (**b**) Comparison of the minimum saturation values between dEES and vEES. The saturations shown are the minimum values all electrode configuration in bipole mode. (**c**) Comparison of the max selectivity index between dEES and vEES. The selectivity index shown are the maximum values all electrode configuration in bipole mode. Data are reported as mean ± SD (a one-sample one-sided t test for bootstrapped samples, n=10,000, *p < 0.05, ***p < 0.001).



**Figure S4.** Simulation results of dEES and vEES in the computational model under tripolar stimulation mode. (a) (b) (c) same as figure S3 for tripole mode.

**Figure S5.** Effects of frequency on the maximum selectivity index and amplitude values under the bipolar stimulation mode. (**a**) Maximum selectivity indices (top) for each muscle under dEES at 10 Hz, 50 Hz, and 100 Hz. Stimulation intensity (bottom) required to achieve the maximum selectivity index at each frequency. (**b**) Same as the (a) for vEES. Data are reported as mean ± SD (one-way ANOVA with Bonferroni correction for bootstrapped samples, n=10,000, *p < 0.05, ***p < 0.001).

**

Figure S6.** Effects of frequency on the maximum selectivity index and amplitude values under the tripolar stimulation mode. (a)(b) same as figure S5 for tripole.
